# Efficient Generation of diRNAs Requires Components in the Posttranscriptional Gene Silencing Pathway

**DOI:** 10.1038/s41598-017-00374-7

**Published:** 2017-03-22

**Authors:** Daisuke Miki, Peiying Zhu, Wencan Zhang, Yanfei Mao, Zhengyan Feng, Huan Huang, Hui Zhang, Yanqiang Li, Renyi Liu, Huiming Zhang, Yijun Qi, Jian-Kang Zhu

**Affiliations:** 1 Shanghai Center for Plant Stress Biology, Shanghai Institutes for Biological Sciences, Chinese Academy of Sciences, Shanghai, 210602 China; 2 Center for Plant Biology, School of Life Sciences, Tsinghua University, Beijing, 100084 China; 3 Department of Horticulture and Landscape Architecture, Purdue University, West Lafayette, IN 47907 USA

## Abstract

It has been reported that double-stranded break (DSB)-induced small RNAs (diRNAs) are generated via the RNA-directed DNA methylation pathway and function in DSB repair in *Arabidposis*. However, important questions remain regarding the biogenesis and function of diRNAs. Here, we used CRISPR/Cas9- or TALEN-triggered DSBs to characterize diRNAs in *Arabidopsis* and rice. We found that 21-nt diRNAs were generated from a 35S promoter::GU-US reporter transgene targeted by CRISPR/Cas9. Unexpectedly, Pol II transcription of the transgene was required for efficient diRNA production and the level of diRNA accumulation correlated with the expression level of the transgene. diRNAs were not detected from CRISPR/Cas9- or TALEN-induced DSBs within the examined endogenous genes in *Arabidopsis* or rice. We also found that DCL4 and RDR6 that are known to be involved in posttranscriptional gene silencing were required to generate diRNAs. Our results suggest that DSBs are necessary but not sufficient for efficient diRNA generation and a high level of diRNAs is not necessary for DSB repair.

## Introduction

Non-coding RNAs play important roles during development and the epigenetic regulation of gene expression in many organisms. Three major small non-coding RNAs have been reported in eukaryotes, microRNAs (miRNAs), small interfering RNAs (siRNAs) and Piwi-interacting RNAs (piRNAs)^1, 2^. MiRNAs are processed from their precursors by Dicer or Dicer-Like (DCL) proteins, and are subsequently incorporated into Argonaute (AGO) family proteins^3^. The miRNA/AGO protein complex negatively regulates gene expression through translation inhibition or target mRNA degradation^3^. siRNAs and piRNAs are derived from transposable elements (TEs) or repetitive sequences to regulate gene expression and defense against exogenous or endogenous invasive genetic elements^3^. In plants, the most abundant siRNA is heterochromatic siRNA (hc-siRNA) which plays a key role in the RNA-directed DNA methylation (RdDM) pathway^4, 5^. In RdDM, single-stranded RNA is transcribed from RdDM target loci by DNA-dependent RNA polymerase IV (Pol IV), synthesized into double-stranded RNA (dsRNA) by RNA-dependent RNA polymerase 2 (RDR2), and then diced into 24 nucleotides (nt) hc-siRNA by DCL3^5^. The hc-siRNA and AGO4/6 complex recognize Pol V transcripts based on homology and recruit *de novo* DNA methyltransferase DRM2 for DNA methylation. Thus, most small RNAs regulate gene expression or maintain genomic/epigenomic stability.

A novel class of small RNAs was recently found to be generated at double-stranded DNA breaks (DSBs), referred to as DSB-induced small RNA (diRNA)^6^. diRNA was originally identified in the GU-US reporter system in *Arabidopsis*, in which the endonuclease I-*Sce*I introduces a DSB at its target site within the linker sequence of GU-US. This DSB is subsequently repaired by homologous recombination, thus restoring β-glucuronidase (GUS) expression and activity^7, 8^. diRNAs accumulated in crosses of the GU-US reporter and I-*Sce*I trigger lines, and were derived from the regions flanking the DSB^6^. A DSB-deficient mutant *atr*, and some RdDM pathway mutants, showed a decrease in DSB repair of the reporter gene and a reduction of diRNA accumulation. Thus, it was thought that DSBs triggered production of diRNAs, and that these diRNAs were required for efficient DSB repair in *Arabidopsis* and human cell lines^6^. Similar small RNA accumulation associated with DSBs was also reported in vertebrate cells, *Droshophila* and *Neurospora crassa* ^9–11^. Further, it was reported that an Ago2/diRNA complex is required to guide the DNA repair factor RAD51 to single stranded DNA filaments at the DSB in human cells^12^. These findings suggest that small RNAs play an important role in the DSB repair pathway^13, 14^.

Recent technological advances have substantially facilitated genome editing approaches in plants and other organisms, even human zygotes^15–20^. These approaches utilize modified nucleases as a tool to edit the genome^21^, including the clustered regularly interspaced short palindromic repeats (CRISPR)/CRISPR-associated protein 9 (Cas9) system and transcription activator-like effector nucleases (TALEN). In the CRISPR/Cas9 system, a single-stranded guide RNA (gRNA) associates with the endonuclease Cas9 and targets it to a specific DNA sequence, thus generating site-specific DSBs^20–22^. On the other hand, TALEN combines “transcription activator-like effectors” (TALE) DNA-binding domains derived from *Xanthomonas* plant pathogens with the *Fok*I nuclease domain^19, 21^. Site-specific DSBs induced by CRISPR/Cas9 or TALEN are subsequently repaired by non-homologous end-joining (NHEJ) or homologous recombination (HR)^21^. The NHEJ pathway is efficient and occurs throughout the entire cell cycle, but is error-prone^21^. During NHEJ, sequence errors are introduced at the DSB sites during DNA repair, which can include deletions or insertions^21^. In contrast, HR is usually error-free and restricted to S and G2 cell cycle phases^21^.

diRNAs have been characterized in transgenes in *Arabidopsis* and human, but the biological relevance of endogenous diRNAs remains largely unknown. Here, we investigated whether diRNAs might function in DSB repair of endogenous genes. We used CRISPR/Cas9 or TALEN to introduce site specific DSBs in *Arabidopsis* and rice. We confirmed that diRNAs were produced in the GU-US transgene, and also in GUS transgenes which lack a direct repeat; however diRNA accumulation was not detected at the endogenous DSBs we tested. Interestingly, Pol II transcription of the transgenes was required for efficient diRNA production, but not for DNA repair. Our findings suggest that efficient diRNA production requires both DSB and active transcription, and DSB repair does not require a high level of diRNAs.

## Results

### diRNAs are generated from transgenes targeted by CRISPR/Cas9

**Figure 1:**
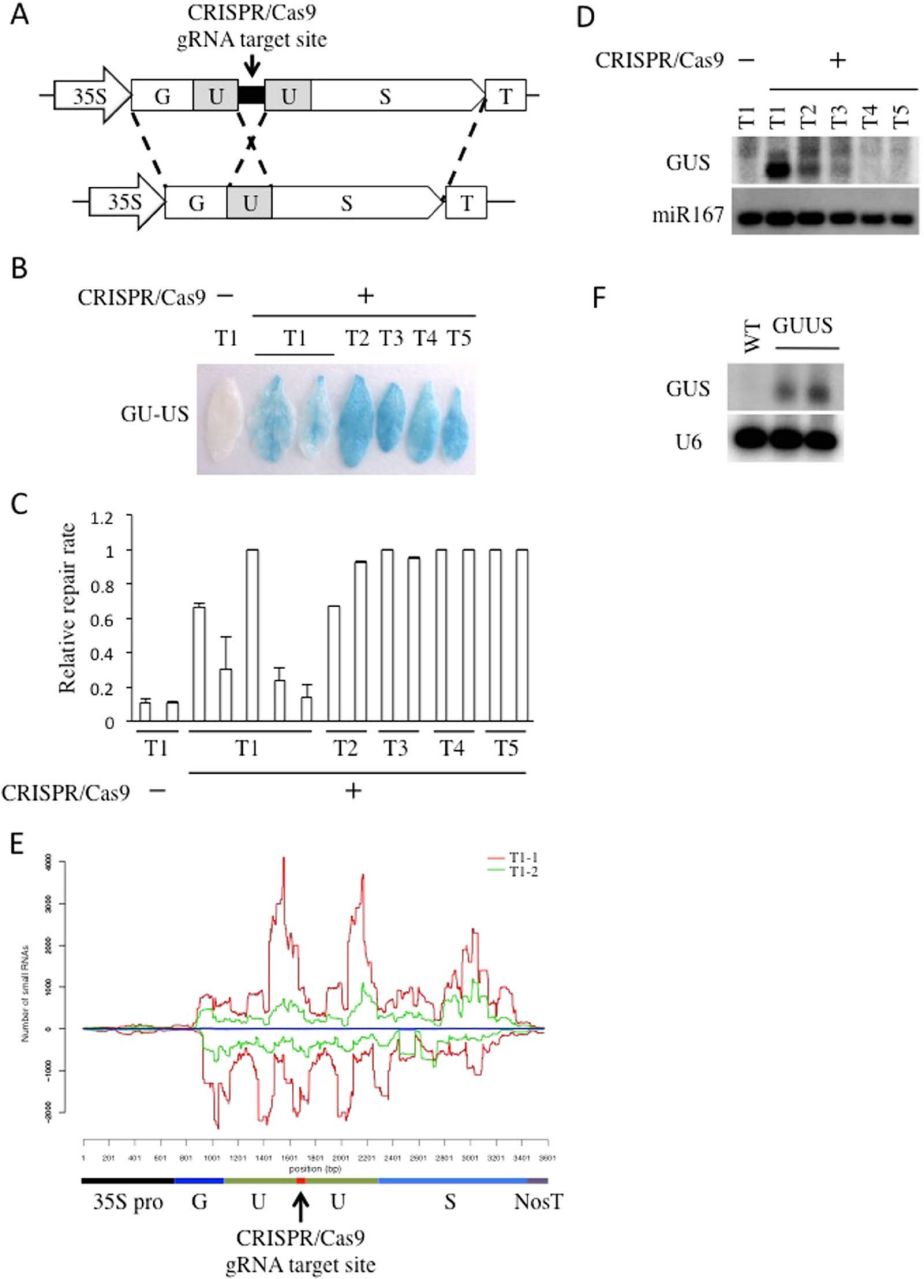
diRNAs are generated from transgenes targeted by CRISPR/Cas9. (**A**) A schematic representation of the 35S::GU-US reporter system. The GU-US reporter contains a CRISPR/Cas9 gRNA target site located within the direct U repeats of GU-US gene. (**B**) Representative GUS staining images of plants containing the GU-US transgene, in the presence or absence of the CRISPR/Cas9 system. The transgenic line that does not carry CRISPR/Cas9 was used as negative control. (**C**) The relative repair rate determined by qPCR. The primers for qPCR amplify the CRISPR/Cas9 target linker sequence and S part of GUS gene, and the repair rate was calculated. Error bars indicate standard error of 3 repeats. (**D**) Detection of small RNAs by Northern blot. The U part of GUS gene was used for the GUS probe. A miR167 probe was used as a loading control. Northern blotting image was cropped nearby signals. (**E**) Distribution of small RNA on 35S::GU-US transgene. Two independent T1 transgenic lines were analyzed. The y axis represents the number of 21 nt long small RNA reads within 100 bp sliding windows with a step size of 1 bp, numbers in (+) and (−) values represent the reads of small RNAs derived from sense and antisense strands, respectively. (**F**) Detection of small RNAs by Northern blot in 35S::GU-US transgenic rice T0 generation. The U part of GUS gene was used for GUS probe. The U6 probe was used as a loading control. Northern blotting image was cropped nearby signals.

It was previously reported that specific DSB-induced RNAs (diRNA) were generated in a transgenic *Arabidopsis* line expressing the I-*Sce*I endonuclease and a DGU.US reporter that contains an I-*Sce*I site^6^. We constructed a similar GU-US reporter system that instead contains a CRISPR/Cas9 single guide RNA (gRNA) target site located within the direct repeats (U) of the non-functional *GUS* gene, and a CRISPR/Cas9 (gRNA/endonuclease) trigger system on the same T-DNA construct (Fig. 1A). The CRISPR/Cas9 complex introduces a single double strand break (DSB) at the target GU-US linker site. This DSB is repaired by homologous recombination (HR) between the direct repeats, which restores functional β-glucuronidase (GUS) (Fig. 1A)^15, 18^. We observed chimeric GUS staining in the T1 generation, and uniform GUS staining in the T2 through T5 generations (Fig. 1B). In contrast, we did not observe visible GUS staining in a transgenic line that expresses the GU-US reporter but not the CRISPR/Cas9 trigger system (Fig. 1B). Thus, CRISPR/Cas9 cleavage followed by HR is necessary to generate a functional GUS gene. Various levels of repaired DNA were detected in independent T1 transgenic lines by qPCR; however, the relative repair rate was almost 100% in generations T2 to T5 (Fig. 1C). The GUS staining and HR repair rate analyses indicated that most CRISPR/Cas9 cleavage and HR repair occured in the T1 and T2 generations, and that, once repaired, the functional GUS gene was stable in subsequent progenies.

It was previously reported that DSBs trigger production of diRNAs form sequences flanking the GU-US DSB sites^6^. We performed Northern blot to examine whether cleavage of the CRISPR/Cas9 target site induced the production of diRNAs from nearby sequences. We used a probe corresponding to the U region of the GUS gene (Fig. 1A), and found that 21 nt small RNAs were scarcely detectable in the GU-US reporter line without CRISPR/Cas9, but were abundant in the T1 generation of the GU-US reporter line with CRISPR/Cas9 (Fig. 1D). The levels of diRNAs corresponding to the U region decreased over subsequent generations, and was hardly detected in the T4 and T5 generations (Fig. 1D). Thus, diRNA production strongly correlated with the timing of GU-US DSB repair. We performed deep sequencing analysis and found that diRNAs that accompany the targeted DSB are distributed across the GU-US transgene (Fig. 1E).

The same 35S::GU-US construct was also transformed into rice to determine if the diRNA production machinery was conserved. We observed a high efficiency of GU-US HR repair in transgenic rice leaves (Supplementary Figure S1), and diRNA accumulation was also detected by Northern blotting (Fig. 1F), indicating that the mechanism underlying diRNA generation is conserved among plant species.

### diRNAs are not generated from endogenous loci targeted by CRISPR/Cas9 or TALEN

**Figure 2:**
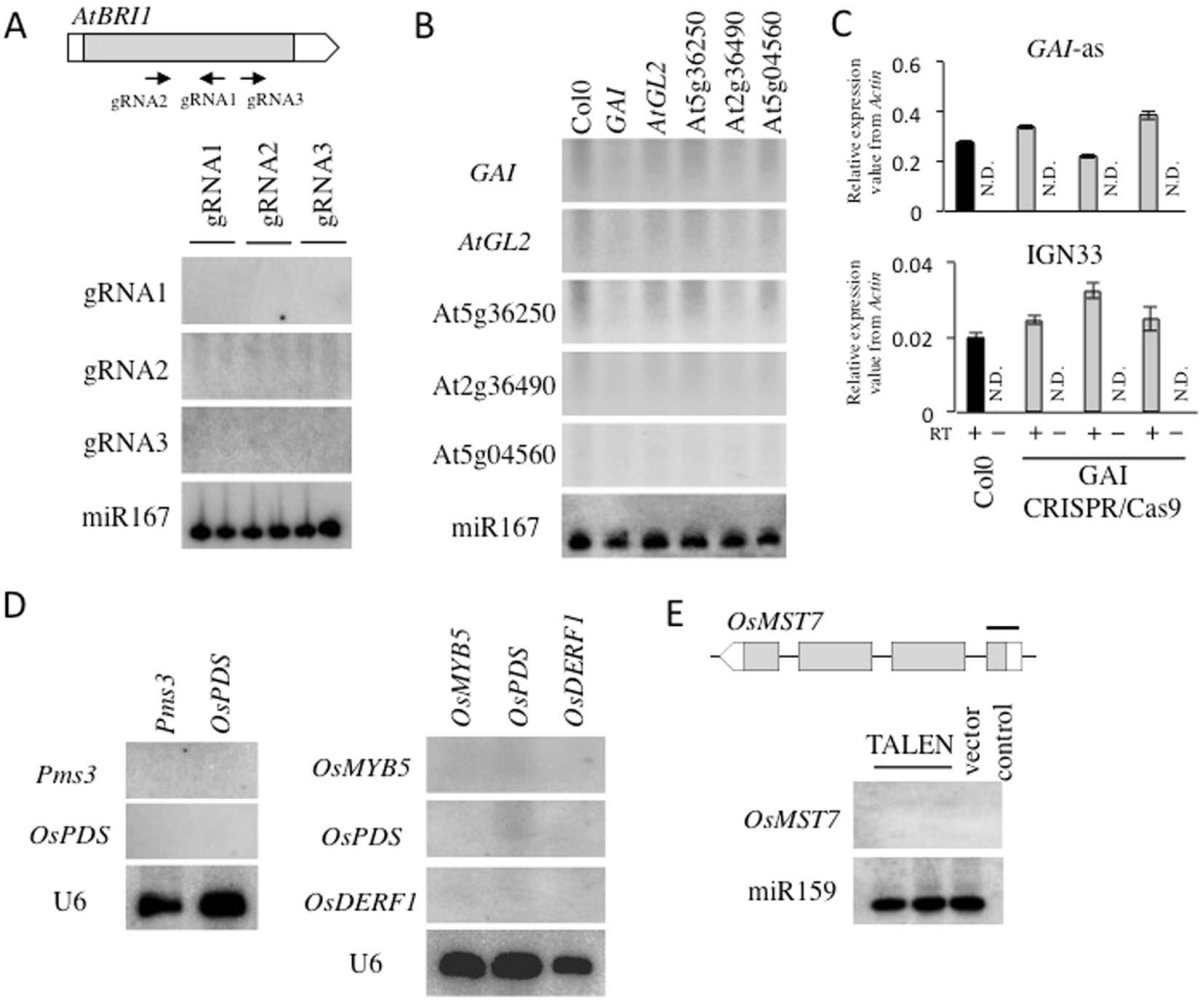
diRNAs are not detected at endogenous genes in *Arabidopsis* and rice. (**A**) Three independent CRISPR/Cas9 constructs target to *AtBRI1*. The top panel is a schematic representation of *AtBRI1* gene and three CRISPR/Cas9 gRNA target sites. Northern blot analysis was performed with LNA probes (see Supplemental Table 1) in *AtBRI1* gene targeted CRISPR/Cas9 T1 transgenic lines. An miR167 probe was used as a control. (**B**) Northern blot analysis of small RNAs for additional endogenous genes (*GAI*, *AtGL2*, At5g36250, At2g36490 and At5g04560) targeted by CRISPR/Cas9. The Col0 accession and T1 transgenic plants were analyzed. (**C**) Detection of antisense transcripts at *GAI* by qRT-PCR, after targeting by CRIPR/Cas9. The same transgenic plants were used as in Fig. 2B. *GAI*-as indicates *GAI* antisense transcript. IGN33 was analyzed as a control. Black bar; Col0 with RT, gray bar; *GAI* targeting CRIPR/Cas9 with RT, white bar; without RT control. N = 3. N.D.; not detected. (**D**,**E**) Detection of small RNAs in endogenous genes targeted by CRISPR/Cas9 (**D**) and TALEN (**E**) in T0 transgenic rice. Horizontal line indicates TALEN target site and probe. U6 and miR159 were probed as controls. (**A**,**B**,**D**,**E**) Northern blotting image was cropped nearby signals.

We next examined whether diRNAs are produced upon the introduction of DSBs within endogenous genes. We used CRISPR/Cas9 transgenic plants and designed three gRNA constructs that target the *BRASSINOSTEROID INSENSITIVE 1* (*AtBRI1*) gene. These three constructs efficiently induced a mutation at *AtBRI1* gene and a visible *AtBRI1* deficient mutant phenotype^16^. We analyzed two transgenic lines for each gRNA construct. Small RNA signals at the corresponding genomic regions within *AtBRI1* were not detectable by Northern blot (Fig. 2A) or deep sequencing (Supplementary Figure S2). We also investigated diRNA generation at other endogenous loci, including *GIBBERELLIC ACID INSENSITIVE* (*GAI*), *AtGL2*, *At5g36250*, *At2g36490* and *At5g04560* (Fig. 2B). Although the gene editing efficiency was high^16^, we did not detect any DSB-induced small RNAs from any of these loci by Northern blotting (Fig. 2B).

It was previously reported that reduced RNA polymerase V (Pol V) activity decreased the HR repair efficiency of GU-US but increased diRNA production^6^. It is known that Pol V generates noncoding RNAs that play important roles in epigenetic modifications^9, 13^. To determine if other noncoding RNAs play a role in HR repair, we evaluated antisense transcripts that might be triggered by CRISPR/Cas9-induced DSBs. We examined T1 transgenic lines in which CRISPR/Cas9 was targeted to *GAI* and found that the level of antisense transcripts produced from the *GAI* locus were comparable in T1 and Col-0 (Fig. 2C). Similarly, we found that the levels of antisense transcripts from four other CRISPR/Cas9-targeted endogenous loci (*AtGL2*, *At5g36250*, *At2g36490* and *At5g04560*) were comparable with Col-0 WT, as determined by qRT-PCR (Supplementary Figure S3).

Next, we determined if CRISPR/Cas9 or TALEN could trigger diRNA production from endogenous loci in rice. The TALE endonuclease, like CRISPR/Cas9, can be used to generate DSBs at target loci efficiently^19, 21^. Five rice genes were chosen as targets of CRISPR/Cas9, and one gene as target of TALEN. We have previously reported that these target genes are efficiently targeted by CRISPR/Cas9 or TALEN^19, 20^. Intriguingly, diRNAs were not detectable in the CRISPR/Cas9 or TALEN transgenic lines by Northern blot (Fig. 2D,E). We also performed deep sequencing and did not detect small RNAs annotated to the targeted gene, *OsMST7*, in TALEN and empty vector control transgenic lines (data not shown).

### diRNAs are not generated from endogenous repeat sequences targeted by CRISPR/Cas9

**Figure 3:**
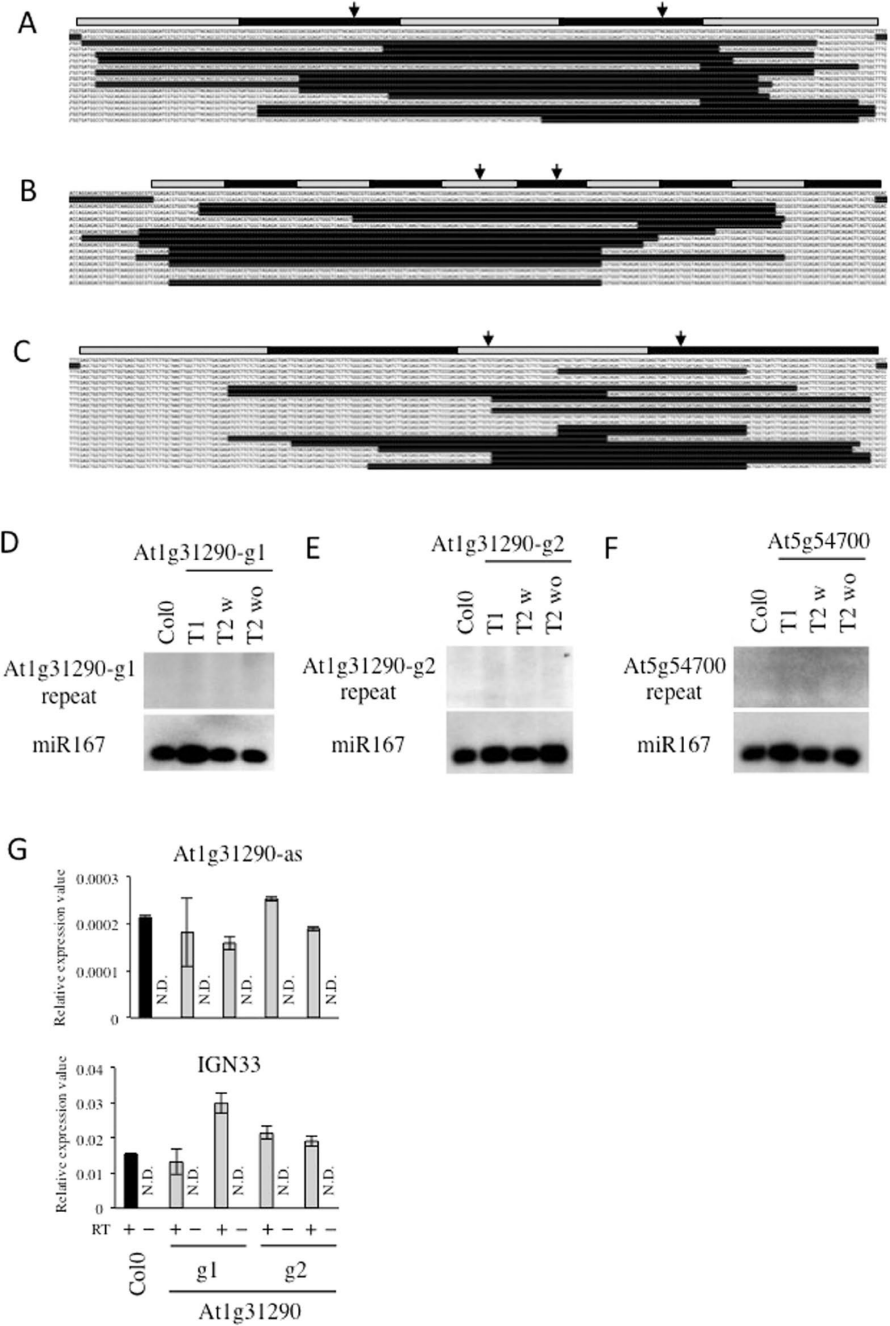
diRNAs are not detected at endogenous repeat regions. (**A**–**C**) The repeat regions and the targeted T1 sequences are shown for the (**A**) At1g31290 repeat1, (**B**) At1g31290 repeat2, and (**C**) At5g54700, respectively. The top gray and black horizontal bars represent the repeats, and the CRISPR/Cas9 target sites are indicated by arrows. The sequence of the repeats is below the gray/black schematic. Below the reference repeat sequence are the sequencing results from independent T1 plants. See also Supplemental Figs 4 and 5. (**D**–**F**) Northern blot analysis for small RNAs in the indicated Col0 control, T1, T2 with CRISPR/Cas9 transgene (T2 w) or without CRISPR/Cas9 transgene (T2 wo). (**D**) At1g31290 repeat1, (**E**) At1g31290 repeat2, and (**F**) At5g54700, respectively. miR167 was probed as control. Northern blotting image was cropped nearby signals. (**G**) qRT-PCR detection of antisense transcripts in At1g31290 repeats targeted by CRISPR/Cas9. At1g31290-as indicates At1g31290 antisense transcript. IGN33 was analyzed as control. Black bar; Col0 with RT, gray bar; At1g31290 targeting CRIPR/Cas9 with RT, white bar; without RT control. N = 3. N.D.; not detected.

Because the GU-US reporter contains a direct repeat and it is also known that DSBs within repeat sequences can induce small RNAs in the filamentous fungus *Neurospora crassa* ^11^, we next investigated if diRNA could be more efficiently generated from endogenous loci containing direct repeats targeted by CRISPR/Cas9. We targeted three distinct endogenous repetitive sequences within the *At1g31290* and *At5g54700* genes using the CRISPR/Cas9 system. *At1g31290* contains two highly homologous but not identical repeats, a 5x repeat of 57 bp and a 10x repeat of 24 bp (Supplementary Figure S4), and *At5g54700* contains a 4x repeat of 72 bp (Supplementary Figure S5). We found that DSBs targeted to these regions by CRISPR/Cas9 induced long deletions (Fig. 3A–C). Direct ligation products of CRISPR/Cas9 target sites were not observed, suggesting that these long deletion might arise from HR-mediated DSB repair^23^. Small RNAs corresponding to these regions did not accumulate in Col-0 plants or in T2 plants that do not express the CRISPR/Cas9 system, as expected; however, small RNAs were also not detected in T1 and T2 plants with targeted DSBs and HR induced by CRISPR/Cas9 (Fig. 3D–F). The levels of antisense transcripts from these repetitive regions were comparable in Col-0 and in T1 plants expressing the CRISPR/Cas9 system, and showed similar variability between plants as a control transcript (Fig. 3G and Supplementary Figure S6).

Taken together, these data suggest that diRNAs and noncoding antisense RNAs are not generated from endogenous genes targeted by CRISPR/Cas9 or TALEN.

### High-level production of diRNAs requires active transcription

**Figure 4:**
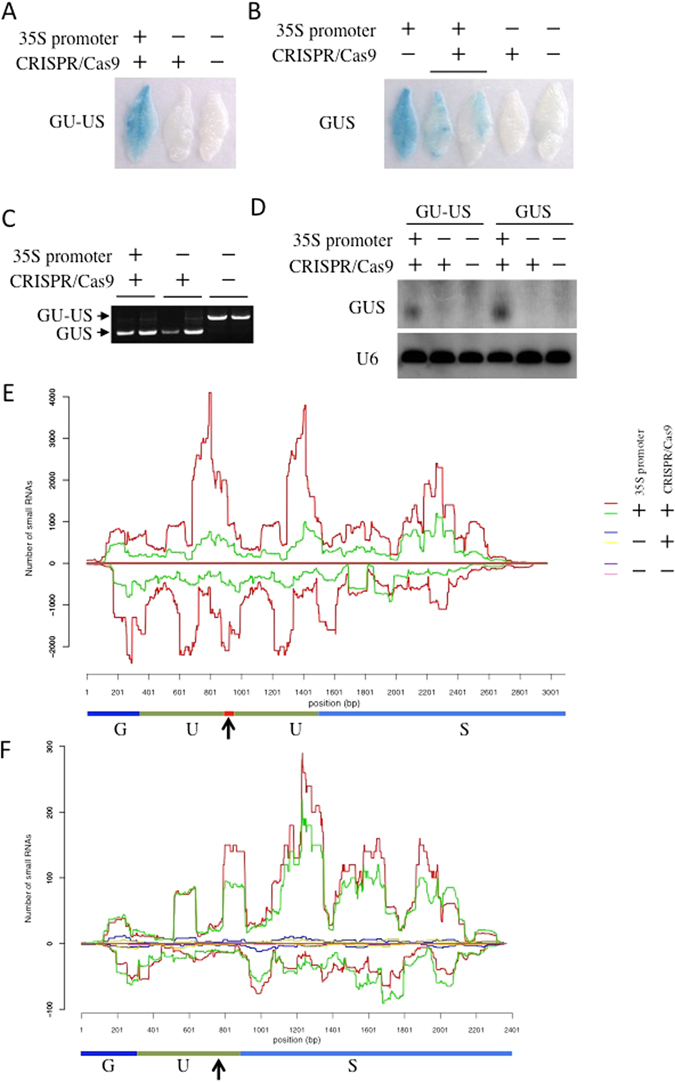
Efficient diRNA production requires active transcription. (**A**) Representative GUS staining images of GU-US reporter system in T1. (**B**) Representative GUS staining images of functional GUS targeted by CRIPSR/Cas9 in T1. (**C**) Detection of GU-US transgene repair by PCR. The longer fragment indicates the parental (unrepaired) GU-US, and the shorter fragment indicates the functional GUS generated by HR. The PCR product size of GU-US and GUS are 1446 bp and 888 bp, respectively. Electrophoresis gel image was cropped nearby signals. (**D**) Detection of small RNAs by Northern blot. The U of GUS gene was as a probe. U6 was probed as control. Northern blotting image was cropped nearby signals. (**E**) The distribution of small RNAs around the GU-US transgene, under the indicated conditions. Two independent T1 transgenic lines for each construct were analyzed. (**F**) The distribution of small RNA for GUS transgene, under the indicated conditions. Two independent T1 transgenic lines for each construct were analyzed. (**E**,**F**) The y axis represents the number of small RNA reads within 100 bp sliding windows with a step size of 1 bp, numbers in (+) and (−) values represent the reads of 21 nt in length small RNAs derived from sense and antisense strands, respectively. The vertical arrows indicate CRISPR/Cas9 target sites. (**A**–**F**) 35S promoter and CRIPSR/Cas9 genotype were indicated as with (+) or without (−).

To determine if HR between the direct repeat U of the GU-US transgene was required to induce diRNAs, we created a transgenic line expressing a 35S::GUS transgene, without repeats, targeted by CRISPR/Cas9. We also created transgenic lines containing promoterless GUS or GU-US transgenes as controls, and found that GUS staining was hardly detected in these transgenic plants with or without the CRISPR/Cas9 system, as expected (Fig. 4A,B). However, we found that the efficiency of HR between the direct U repeats was similar in plants with or without 35S promoter (Fig. 4C and Supplementary Figure S7), suggesting that transcription of the GU-US transgene is not necessary for efficient HR. We found that GUS staining was reduced in lines containing the 35S::GUS transgene with the CRISPR/Cas9 system, compared to those without CRISPR/Cas9 (Fig. 4B). This result suggested that some mutations were introduced into the GUS gene by CRISPR/Cas9, which we confirmed by sequencing (data not shown).

We analyzed small RNA accumulation in transgenic lines containing GU-US or GUS transgenes, with and without CRIPR/Cas9 that induces DSBs. We detected abundant diRNAs in both the 35S::GU-US and 35S::GUS transgenic lines containing the CRISPR/Cas9 system by Northern blot (Fig. 4D). This indicates that HR between the direct U repeats was not necessary to generate diRNAs, and that a DSB in 35S::GUS is sufficient for diRNA production. Intriguingly, diRNA signals were hardly detected from either of the promoterless GU-US and GUS transgenic lines expressing CRISPR/Cas9 (Fig. 4D). These Northern blot results were confirmed by small RNA deep sequencing analysis (Fig. 4E,F).

We examined if the expression level of the 35S::GU-US transgene correlated with the level of diRNA accumulation. We used Northern blot to analyze diRNA accumulation in 15 independent T1 lines categorized into four groups based on the expression level of the 35S::GU-US. Although the HR repair efficiency was comparable among the four groups, we found that diRNAs accumulated only in Group 1, which expressed the highest levels of the 35S::GU-US transgene (Supplementary Figure S8). These results further suggest that abundant Pol II transcripts of the GU-US transgene are necessary for diRNA production.

Considered together, these results suggest that efficient generation of diRNAs requires active transcription of the CRISPR/Cas9 target region.

### Genetic requirements for diRNA generation

**Figure 5:**
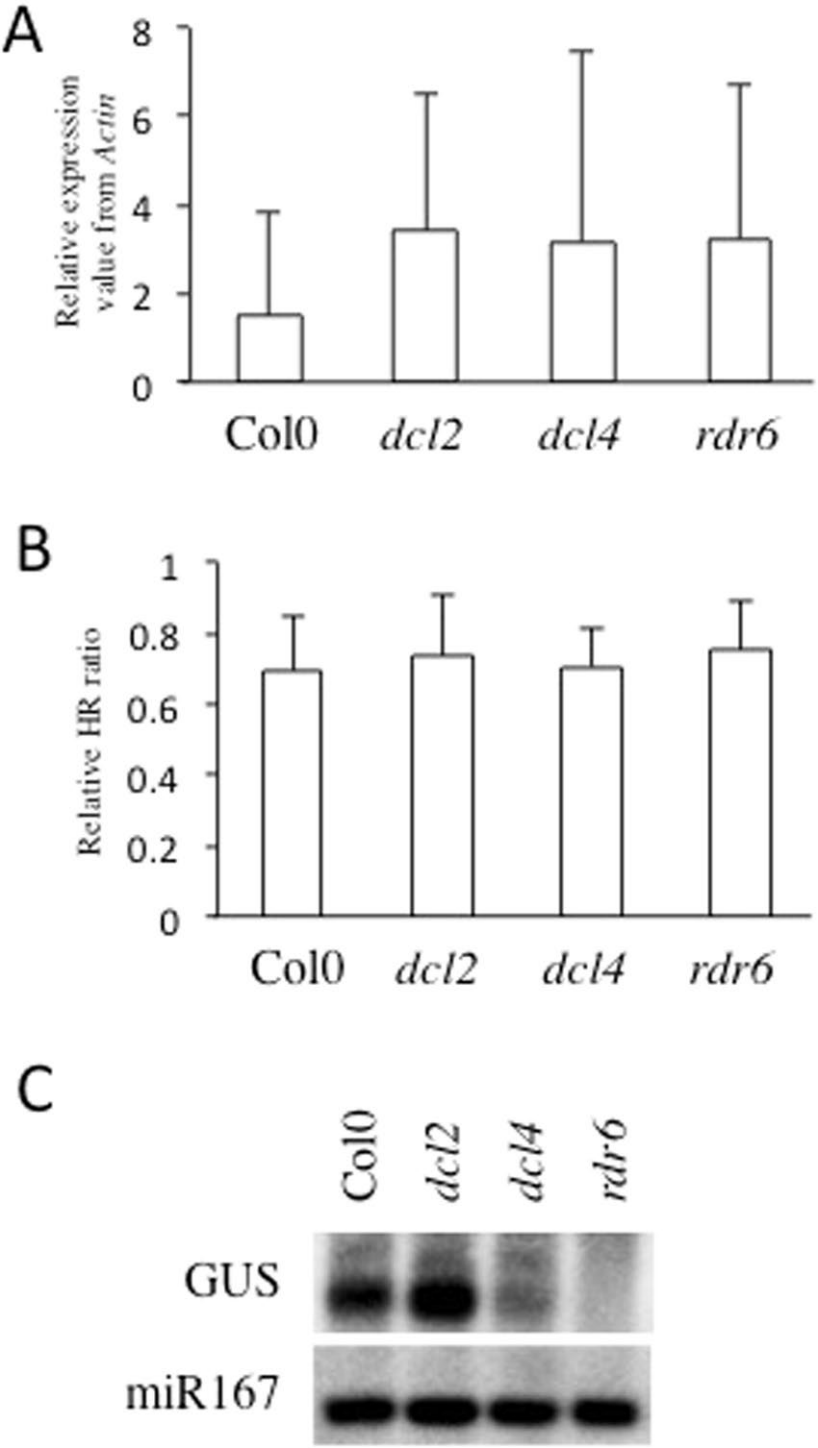
Genetic requirements for efficient diRNA generation. (**A**) GU-US transgene expression in PTGS mutants. Relative expression was determined by qRT-PCR in PTGS mutants. Error bars indicate standard error of 5 independent T1 transgenic lines. (**B**) Relative HR rate determined by qPCR. Error bars indicate standard error of 5 independent T1 transgenic lines. (**C**) Detection of small RNAs. The U part of GUS gene was used as a probe. miR167 was probed as a loading control. Northern blotting image was cropped nearby signals.

The characteristics of diRNAs that we have uncovered in this study, including a length of 21 nt and an association with highly transcribed transgenes but not endogenous genes, are similar to those of secondary siRNAs in the post-transcriptional gene silencing (PTGS) pathway^24^. To investigate the roles of these pathways in the generation of diRNAs, we transformed the 35S::GU-US construct into *dcl2*, *dcl4* and *rdr6* mutants. We determined the expression levels of the 35S::GU-US transgene in each of the independent T1 transgenic lines by qRT-PCR, and five of the highly expressed lines from each genetic background were analyzed further (Fig. 5A). We found that the efficiency for HR-mediated repair of 35S::GU-US was similar among the different genetic background (Fig. 5B). Further, DSB-induced small RNAs corresponding to GU-US were reduced in the *dcl4* and *rdr6* mutants, but not in *dcl2* mutants (Fig. 5C). Thus, genes required for PTGS, DCL4 and RDR6, are necessary for diRNA accumulation, but not for efficient HR-mediated repair of the GU-US transgene.

To investigate potential roles for the RdDM pathway in diRNA generation and HR, we created transgenic lines expressing the GU-US target transgene or the CRISPR/Cas9 system in RdDM mutants, and used these to generate the F1s for analysis. We used 16–27 independent F1 lines to analyze the efficiency of HR-mediated repair of GU-US by qPCR. The HR efficiency was decreased in *nrpd1* and *nrpe1* mutants, but not drastically (Supplementary Figures S9 and S10), consistent with a previous report^6^. Interestingly, the HR efficiency was slightly enhanced in the *ago4/6* double mutant and *nrpd2* mutant, and substantially enhanced in the *ago1* mutant (Supplementary Figures S9 and S10). AGO2 was previously reported to be a diRNA-interacting Argonaute protein required for efficient HR repair^6^. In our experiment, however, the HR efficiency was not altered in the *ago2* mutant (Supplementary Figures S9 and S10). The increase in HR efficiency associated with the *ago1* mutant suggests that miRNAs may influence DSB repair. It was previously reported that miRNA transcription and maturation processes respond to DNA damage in human cell lines, and some miRNAs negatively regulate DSB repair core components^25, 26^. Similar mechanisms may also exist in plants to suppress DSB repair.

We also analyzed the expression of the GU-US and Cas9 transgenes in the RdDM mutant lines by qRT-PCR. The expression of both transgenes was elevated in *ago4/6* and *nrpe1* mutants, and suppressed in the other mutants (Supplementary Figure S9). Both transgenes are transcribed from the 35S promoter; the altered expression levels might arise from epigenetic changes at this promoter in different mutant backgrounds^5, 27^. Next, we investigated the accumulation of diRNAs by Northern blotting. The levels of diRNA were similar between Col-0, *ago2*, *ag4/6* and *nrpd1*, but highly elevated in *nrpe1* mutant (Supplementary Figure S9). The increased diRNA signal in *nrpe1* mutant may be due to derepression of the 35S::GU-US transgene. Together, these results suggest that the RdDM pathway plays a minor role, if any, in diRNA production and efficient HR repair.

## Discussion

We have shown that diRNAs are generated at CRISPR/Cas9-induced DSBs within a GU-US transgene in *Arabidopsis*, consistent with previous results^6^. Further, we found that diRNAs were also generated at targeted DSBs within a GUS transgene that lacks a direct “U” repeat, indicating that homologous recombination was not necessary for diRNA production. These data suggest that diRNAs are associated with DSBs, but not a specific repair pathway. We also found that not all DSBs generate diRNAs, in particular we could not detect diRNAs at the endogenous genes or repetitive sequences that we targeted by CRISPR/Cas9 and TALEN in *Arabidopsis* and rice. Thus, DSBs appear to be necessary but not sufficient to generate diRNAs.

We also observed that diRNA production required not only DSBs, but also transcription of the region containing the DSB. Indeed, the level of transcription correlated with the level of diRNAs. In contrast, the efficiency of HR- or NHEJ-mediated repair was similar in the presence or absence of diRNAs. Thus, diRNAs do not seem to play a role in DNA repair, and may be a consequence of high transcription together with DSBs, which may lead to aberrant transcripts arising from inappropriate processing of the transcripts at the DSB site.

We showed that diRNAs, which are 21 nt long, require DCL4 and RDR6 for their biogenesis, and associate with the expression of the transgenes, but not the endogenous genes, we tested. These characteristics resemble secondary small RNAs of the PTGS machinery. Secondary small RNAs are a product of transitive RNA silencing, which has been reported in plants, nematodes, fungi and bacteria^24, 28–33^. The secondary small RNAs amplify silencing caused by a primary siRNA, and require RNA dependent RNA-polymerase (RDR) activity for their biogenesis. In *Arabidopsis*, RDR6 uses cleaved mRNAs as a template for dsRNA synthesis, and DCL4 generates 21 nt long secondary small RNAs from these dsRNAs^31, 34, 35^. During the amplification step, RDR6 synthesizes dsRNAs that extend beyond the 5′ and 3′ boundaries of the initial primary siRNA target site. Transitive RNA silencing has been associated with transgenic transcripts, but rarely with endogenous transcripts^24, 32, 36, 37^. One of the big differences between the transgenes and endogenous genes is their transcript levels. For highly accumulated endogenous mRNAs, some small RNAs may be produced from them through PTGS pathway^38, 39^. Thus the lack of diRNAs from endogenous genes in this study could be due to their low rate of transcription.

While our work confirmed previous reports on the existence of diRNAs from transgenes^6^, our results on the genetic requirements for diRNA generation and on the relationship between diRNAs and DSB repair differ from the previous work. The differences could be attributed to the different experimental systems employed by these studies.

We propose a model of diRNA production in Supplementary Figure S11. In this model, DSBs, induced by CRISPR/Cas9 or a restriction endonuclease, trigger transcription of an aberrant mRNA that is recognized by RDR6 and/or other RDRs. Primary siRNAs generated by RDR6 and DCL2/4 recognize the aberrant and intact transgene mRNA, and thus promote the biogenesis of secondary siRNAs (Supplementary Figure S11). The target mRNA cleavage activity of secondary siRNAs is lower than that of primary siRNAs^29, 40^, which could explain why the target transgene mRNA and secondary siRNAs were concomitantly detected. Our findings suggest that the diRNAs we have described herein are not required for efficient DSB repair. Instead, our data suggest that diRNAs are equivalent to small RNAs of the PTGS pathway, and that DSBs at certain transgenes could trigger PTGS via the production of aberrant transcripts.

Some long non-coding RNAs have been reported that respond to genotoxic stresses^41–43^. Further, Pol V generates long non-coding RNAs, and it was reported that HR efficiency was reduced in a Pol V mutant background^6^. A working model was proposed in which Pol V transcribes non-coding RNA from the vicinity of DSB sites, and this non-coding RNA would serve as scaffold for an AGO2/diRNA complex to promote efficient DSB repair^6, 14^. Thus, we expected that non-coding/antisense RNAs might be transcribed at the CRISPR/Cas9 target sites. However, the level of antisense transcripts at endogenous target loci were comparable with WT, suggesting that neither diRNAs nor non-coding/antisense RNAs are required for efficient DSB repair at the examined loci.

We would like to propose two possibilities. The diRNAs detected in this study and previous studies^6^ may constitute primary and secondary diRNAs. Primary diRNAs may be produced from yet-to-be repaired DSB sites and may not require high-level transcription for their production. They are of low abundance but play a role in DSB repair. Secondary diRNAs are produced from transcripts from the repaired DNA and require active transcription for their production. They are more abundant but might not contribute to DSB repair. Another possibility is that the diRNAs we have described herein are not required for efficient DSB repair. Instead, our data suggest that diRNAs are equivalent to small RNAs of the PTGS pathway, and that DSBs at certain transgenes could trigger PTGS via the production of aberrant transcripts.

## Materials and Methods

### Gene accession numbers

*AtBRI1*; At4g39400, *GAI*; At1g14920, *Ago3*; At1g31290, *OsPDS*; Os03g08570, *OsMYB5*; Os05g41166, *OsDERF1*; Os08g35240, *OsMST7*; Os01g38680.

### Plant materials and constructs

*Arabidopsis* Col-0 accession was used for all experiments. All plants were grown on Murashige and Skoog (MS) medium or soil at 16 hour light/8 hour dark photoperiod. CRIPSR/Cas9 and TALEN constructs were described previously^15, 16, 18–20^.

### GUS staining

For GUS staining, cotyledons or 4-week-old leaves were collected and infiltrated in 0.5 mg/ml X-gluc (Gold Bio COM), 50 mM sodium phosphate (pH 7.0), 10 mM EDTA buffer, followed by incubation at 37 °C overnight. Leaves were cleared in ethanol.

### DNA analysis

Total DNA was extracted by the cethyltrimethyl ammonium bromide (CTAB) method from 4-week-old mature leaves. Extracted DNA was used to analyze the relative repair rate by PCR and qPCR, and in sequencing to detect mutations. To identify mutations, PCR products were cloned into pMD18-T vector (Takara), and at least ten independent clones were sequenced. To detect GU-US and GUS, PCR products were run on 1% agarose gel for 100 V 30 min, and visualized by Image Lab Software and Gel Doc XR (BIO-RAD). Primers used for these PCR reactions are listed (Supplementary Table S1).

### Calculation of GU-US HR ratio

The primers for qPCR amplify the CRISPR/Cas9 target linker sequence of GU-US and the S region of GUS, using extracted DNA as a template. The repair rate was calculated by using the qPCR values in the following equation: (S region of GUS – linker of GU-US)/S region of GUS.

### RNA analysis

Total RNA was extracted from 4-week-old mature leaves with Trizol reagent (Invitrogen). 50 μg of RNA enriched for small RNAs was separated by acrylamide gel electrophoresis, followed by Northern blot analysis according to published protocols^44^. To detect small RNAs at the target regions, a PCR fragment was labeled with 32P-α-dCTP by using the Random primer DNA labeling kit (Takara). The miR167, miR159, U6 and LNA were probed with end-labeled oligonucleotides by T4 polynucleotide kinase (NEB). Northern blot signals were detected with a phosphor imager (Fuji). For RT- and qRT-PCR, total RNA was treated with Turbo DNA-free (Ambion) and reverse transcribed by TransScript II (TransGen Biotech) with gene specific primers or oligo (dT) primer. For detection of antisense transcripts, 2 pmol of forward primer was used for reverse transcription reaction in 20 μl volume. The sequences of the primers and oligonucleotides are listed (Supplementary Table S1).

## Electronic supplementary material

Supplementary Information

Supplementary Table
