# Supplementary material for: Efficient Generation of diRNAs Requires Components in the Posttranscriptional Gene Silencing Pathway
Source: Sci Rep. 2017 Mar 22;7:301. doi: 10.1038/s41598-017-00374-7 (PMC5428250; doi:10.1038/s41598-017-00374-7)
Supplement: Supplementary file 1 — Supplementary Information [file 41598_2017_374_MOESM1_ESM.pdf]

Efficient Generation of diRNAs Requires Components in the Posttranscriptional Gene Silencing Pathway

Daisuke Miki[1 +*], Peiying Zhu[1 +], Wencan Zhang[1 +], Yanfei Mao[1], Zhengyan Feng[1], Huan Huang[1], Hui Zhang[1], Yanqiang Li[1], Renyi Liu[1], Huiming Zhang[1], Yijun Qi[2] and Jian-Kang Zhu[1,3]

AFFILIATIONS

[1]Shanghai Center for Plant Stress Biology, Shanghai Institutes for Biological Sciences, Chinese Academy of Sciences, Shanghai 210602, China

[2]Center for Plant Biology, School of Life Sciences, Tsinghua University, Beijing 100084, China

[3]Department of Horticulture and Landscape Architecture, Purdue University, West Lafayette, IN 47907, USA

[+] These authors contribute equally to this work

[*] To whom correspondence should be addressed.

Email: Miki, Daisuke (miki@sibs.ac.cn)

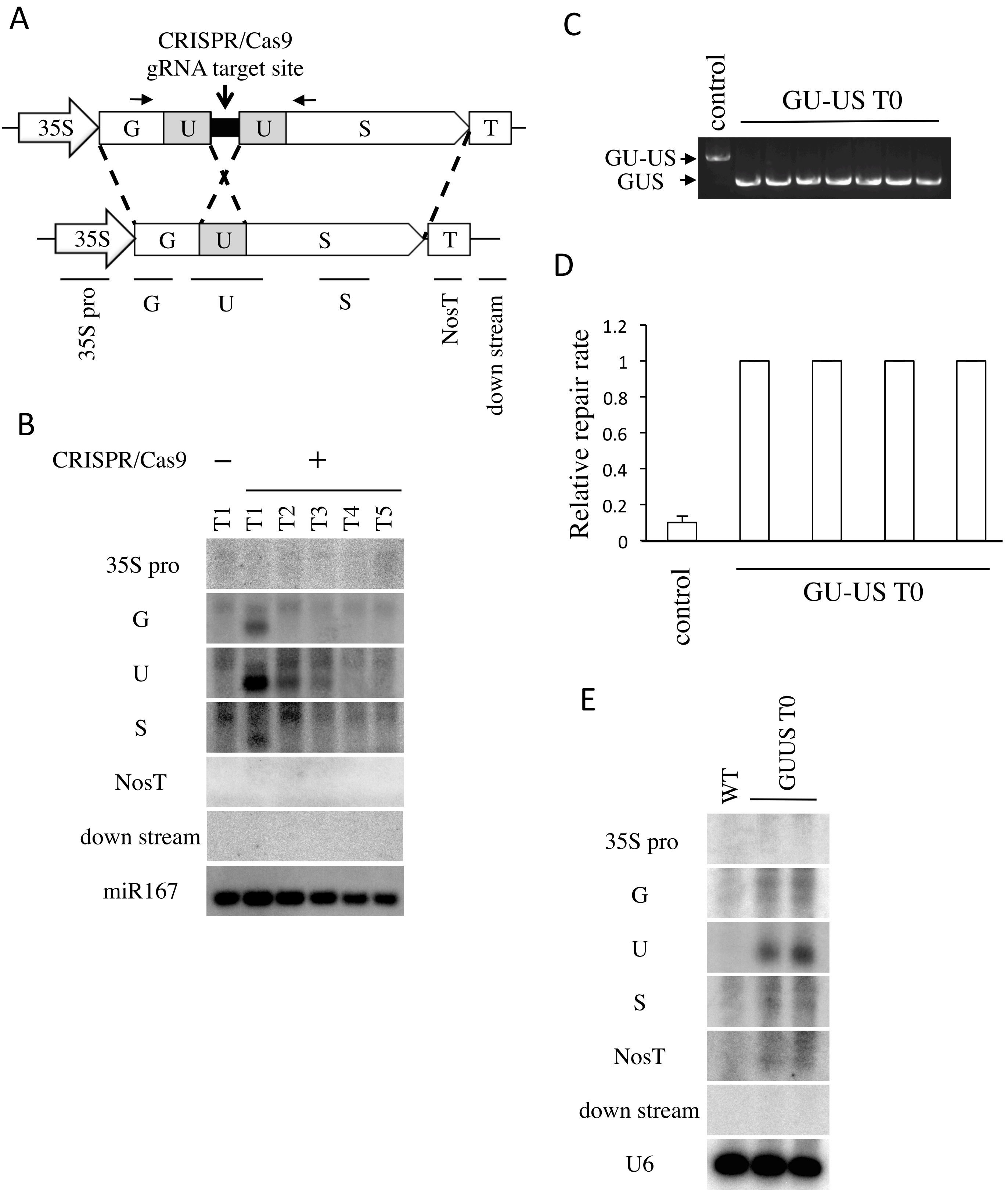

Supplementary Figure S1. small RNA Northern blotting for the GU-US transgene in *Arabidopsis* and rice

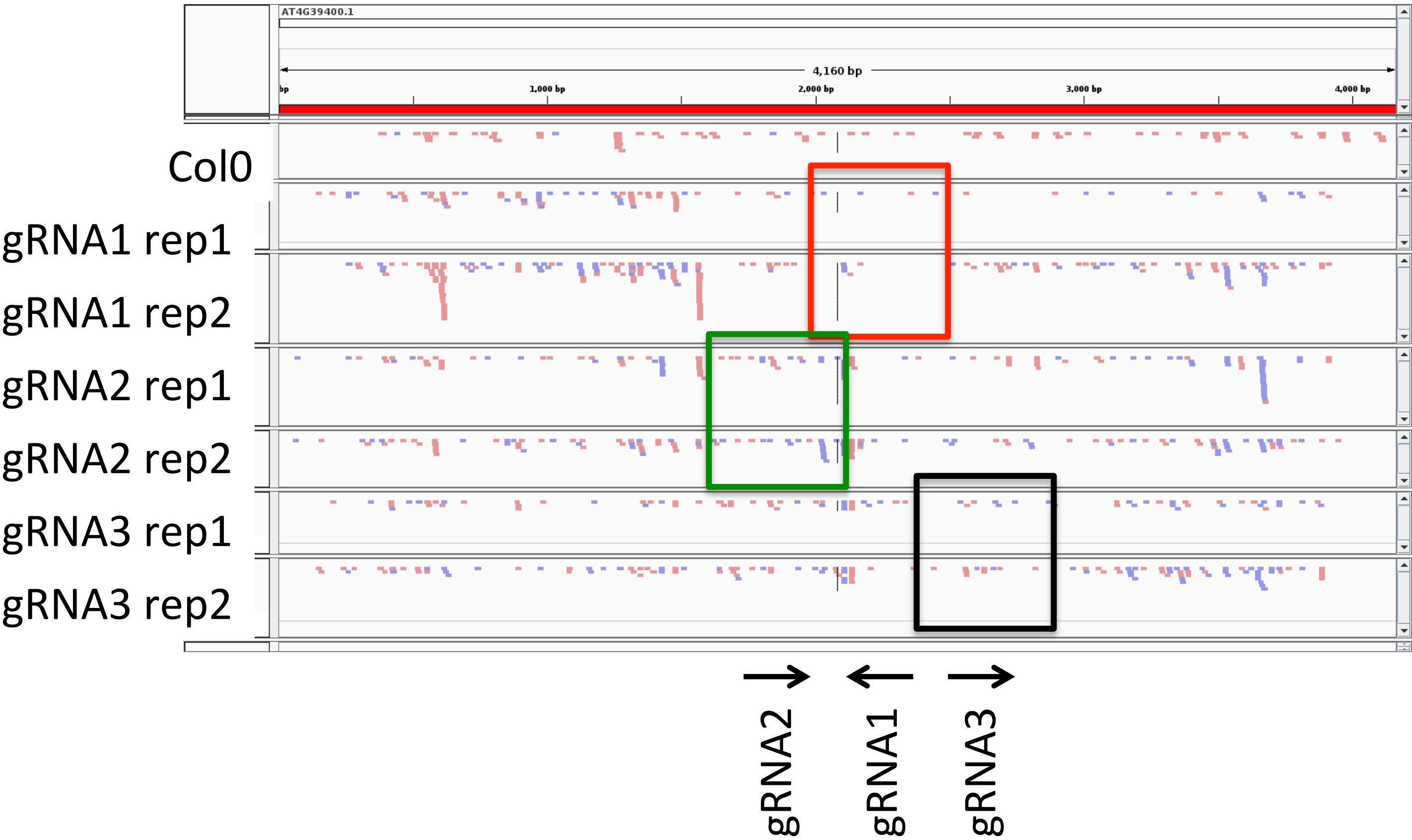

Supplementary Figure S2. Deep sequencing of small RNA analysis for *AtBRI1* targeted CRISPR/Cas9 transgenic lines

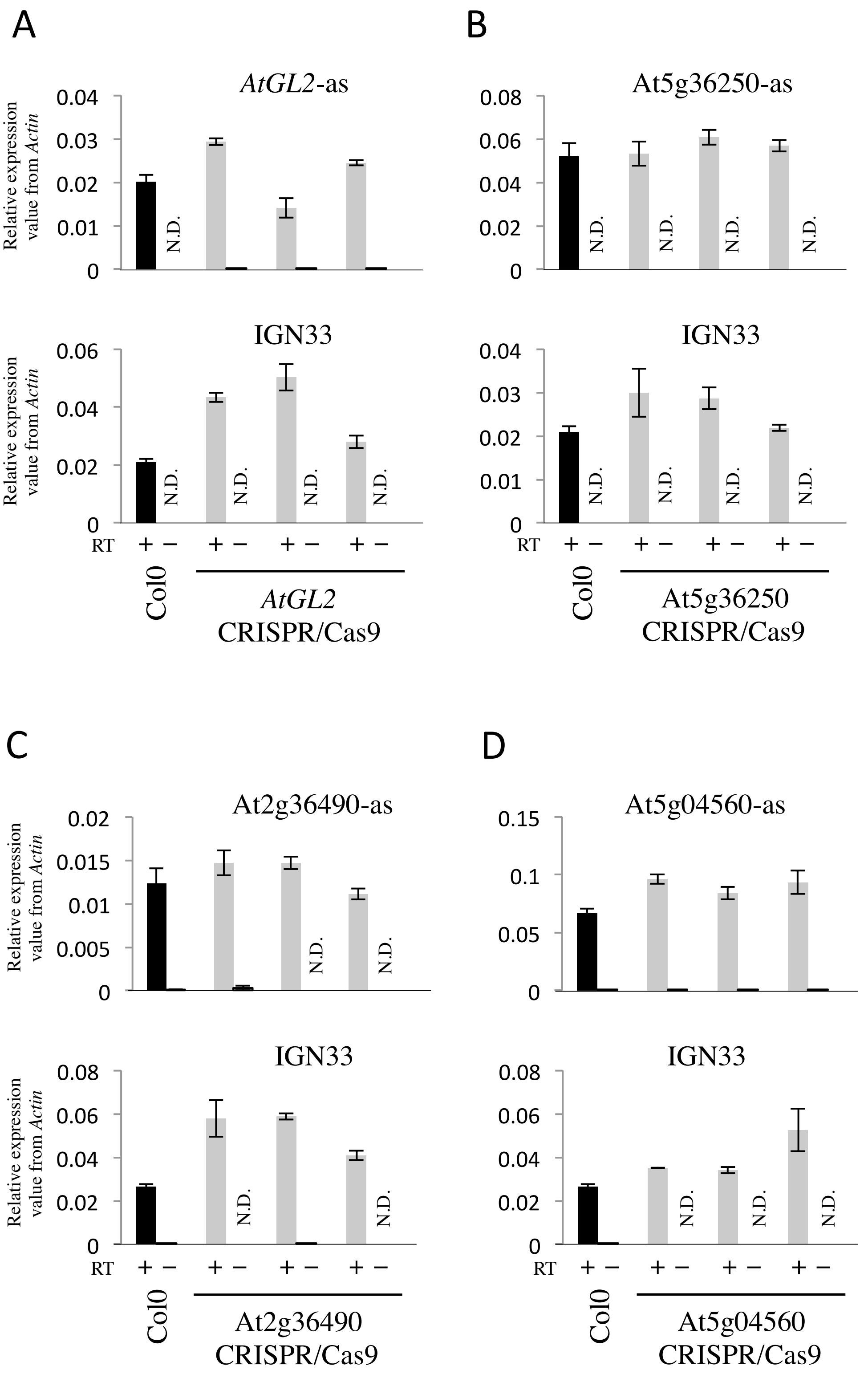

Supplementary Figure S3. Antisense transcript analysis by qRT-PCR

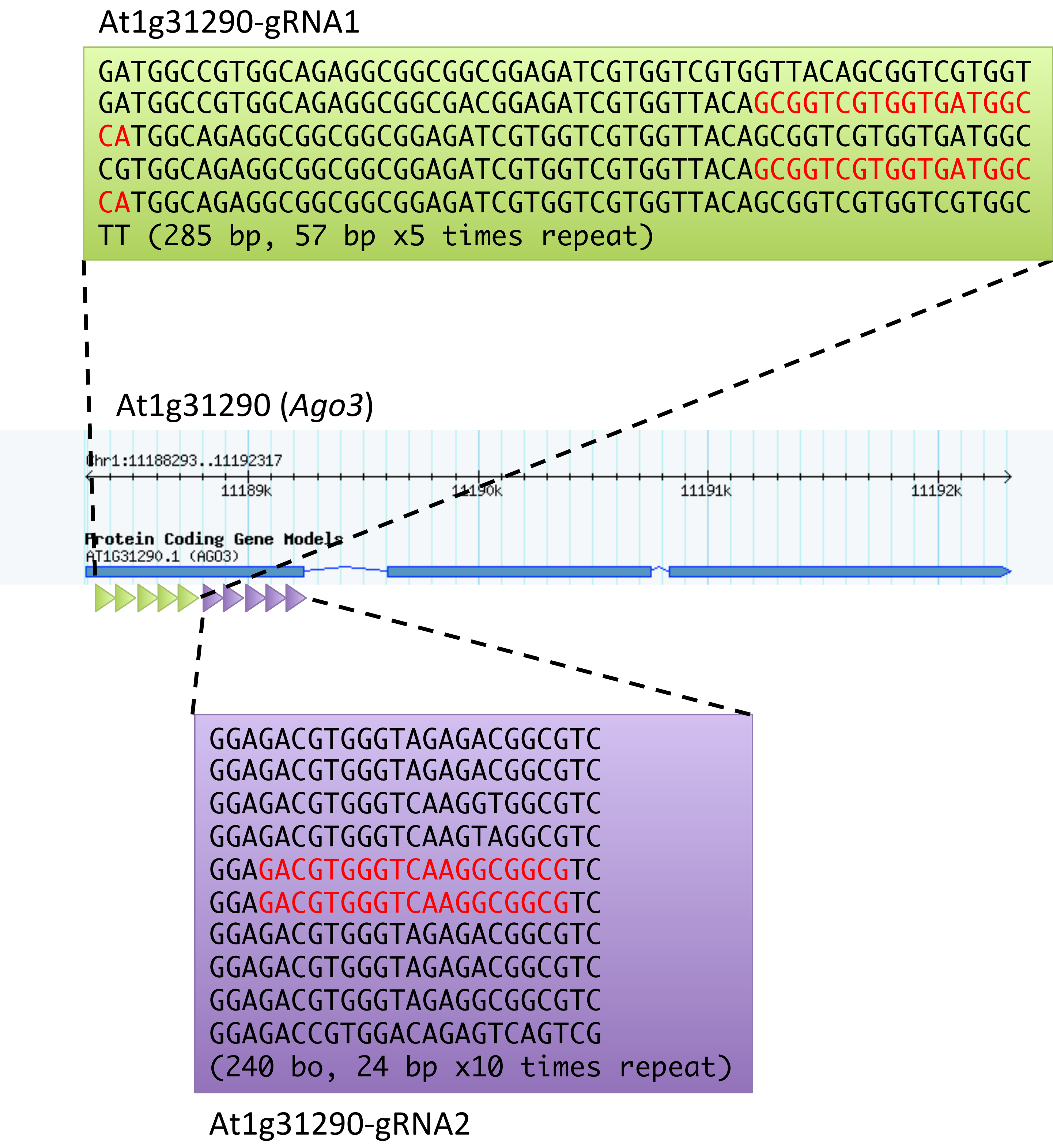

Supplementary Figure S4. CRISPR/Cas9 targeting to endogenous repeat regions in the At1g31290 gene

At5g54700

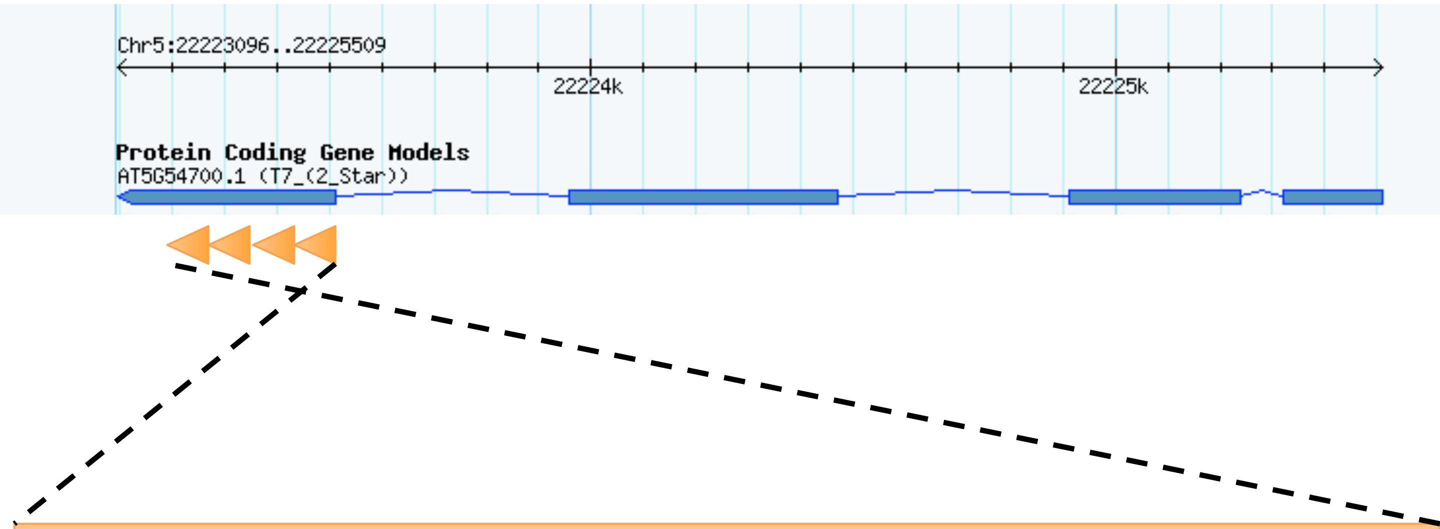

GAGCTGGTGGTTCTGG-TGAGCTGGCTCTTCTTGCTAAGTTGGCTTGTCTTGACGAGATGTCTTCTCTCGAC
GAGCTGACTTGTACCGATGAGCTGGCTCTTCTGGGCGAGCTGGCTGATCTTGACGAGCAGACTTCTCCCGAC
GAGCTGACTTGTGTCGATGAGCTGGCTCTTCTGGGCGAGTTGGCTGATCTTGACGAGCAGACTTCTCCCGAC
GAGCTGACTTGTGTCGATGAGCTGGCTCTTCTGGGCGAACTGGCTGATCTTGACGAGCAGACTTCTCCCGAC
GAGCTGACTTGTGCT (303 bp, 72 bp x4 times repeat)

Supplementary Figure S5. CRISPR/Cas9 targeted to an endogenous repeat region in the At5g54700

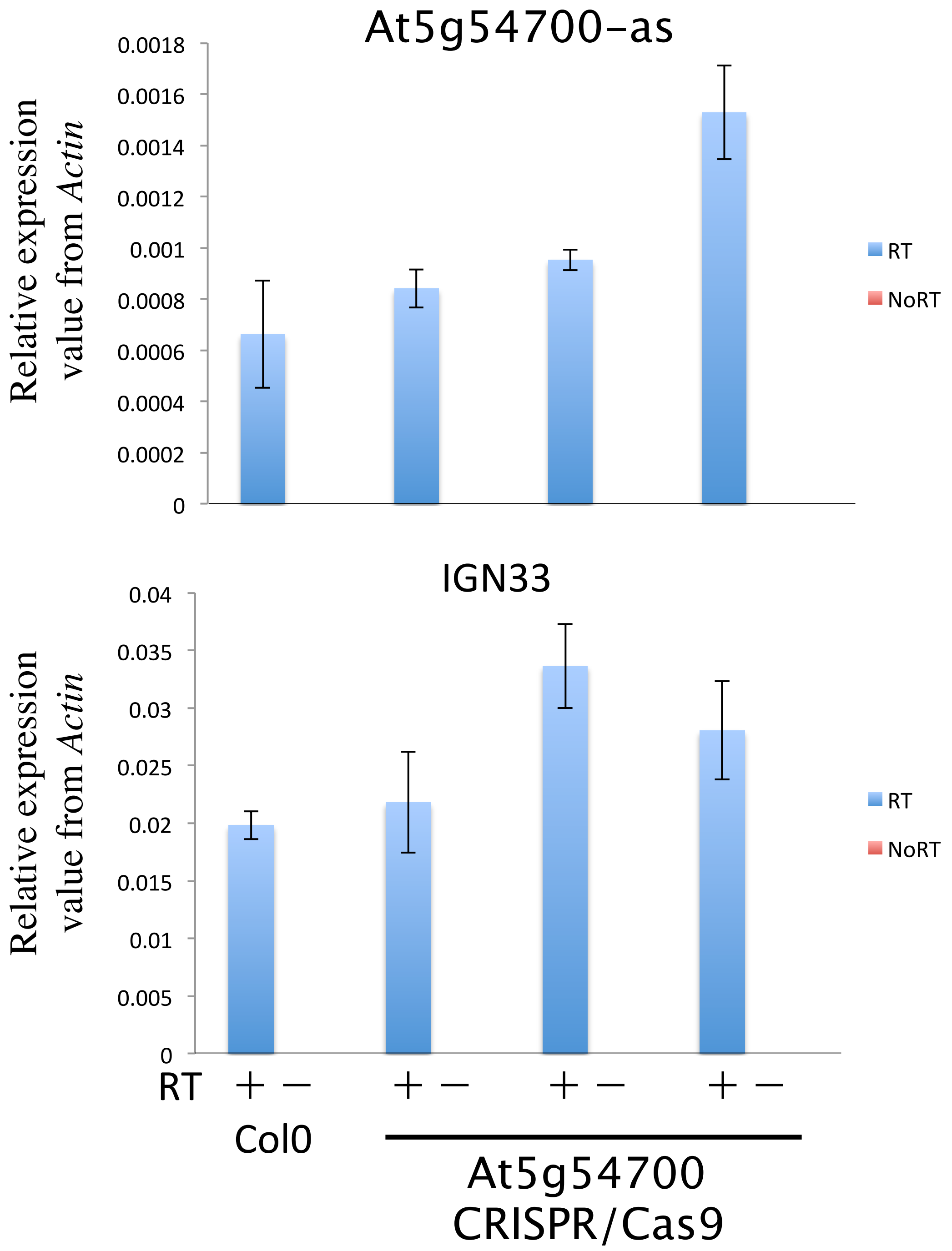

Supplementary Figure S6. Detection of antisense transcript from At5g54700 repetitive region

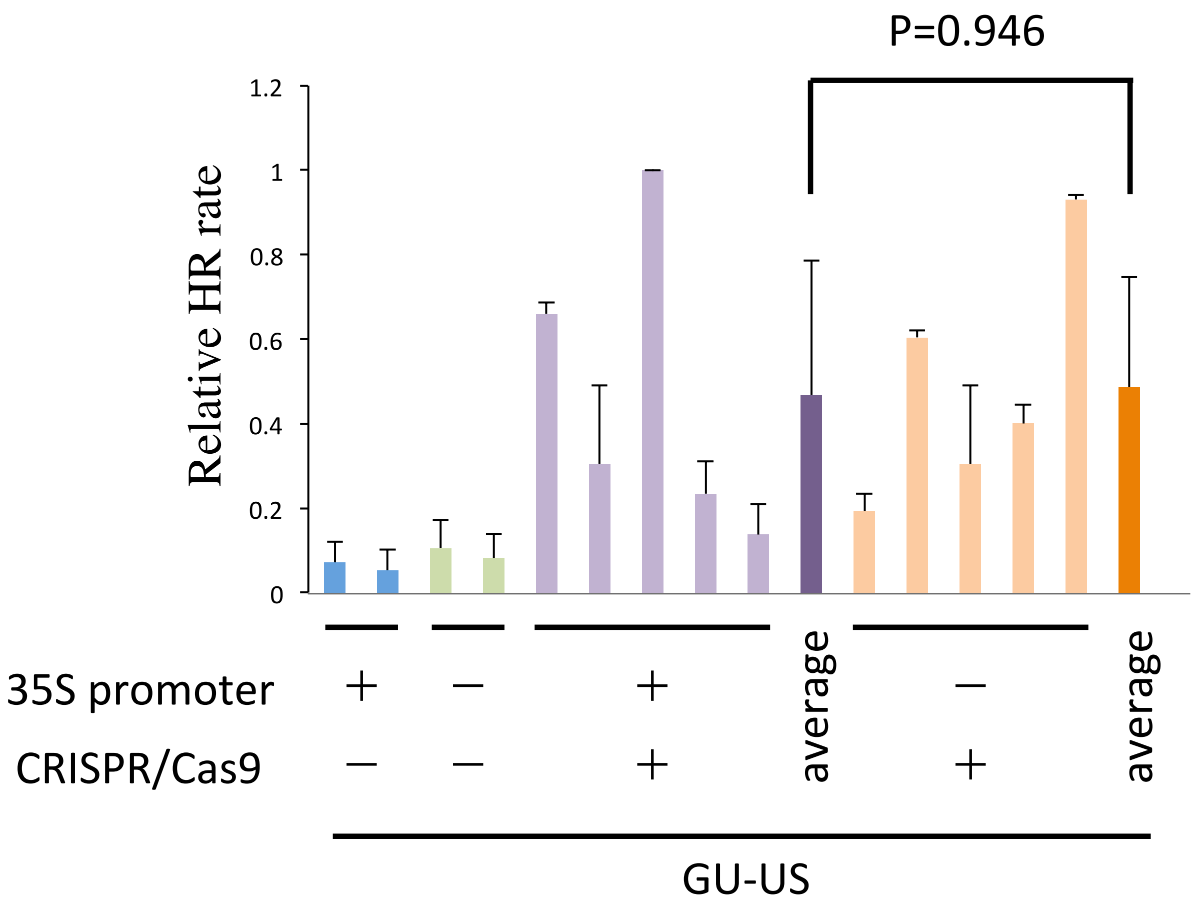

Supplementary Figure S7. qPCR analysis for GU-US repair ratio

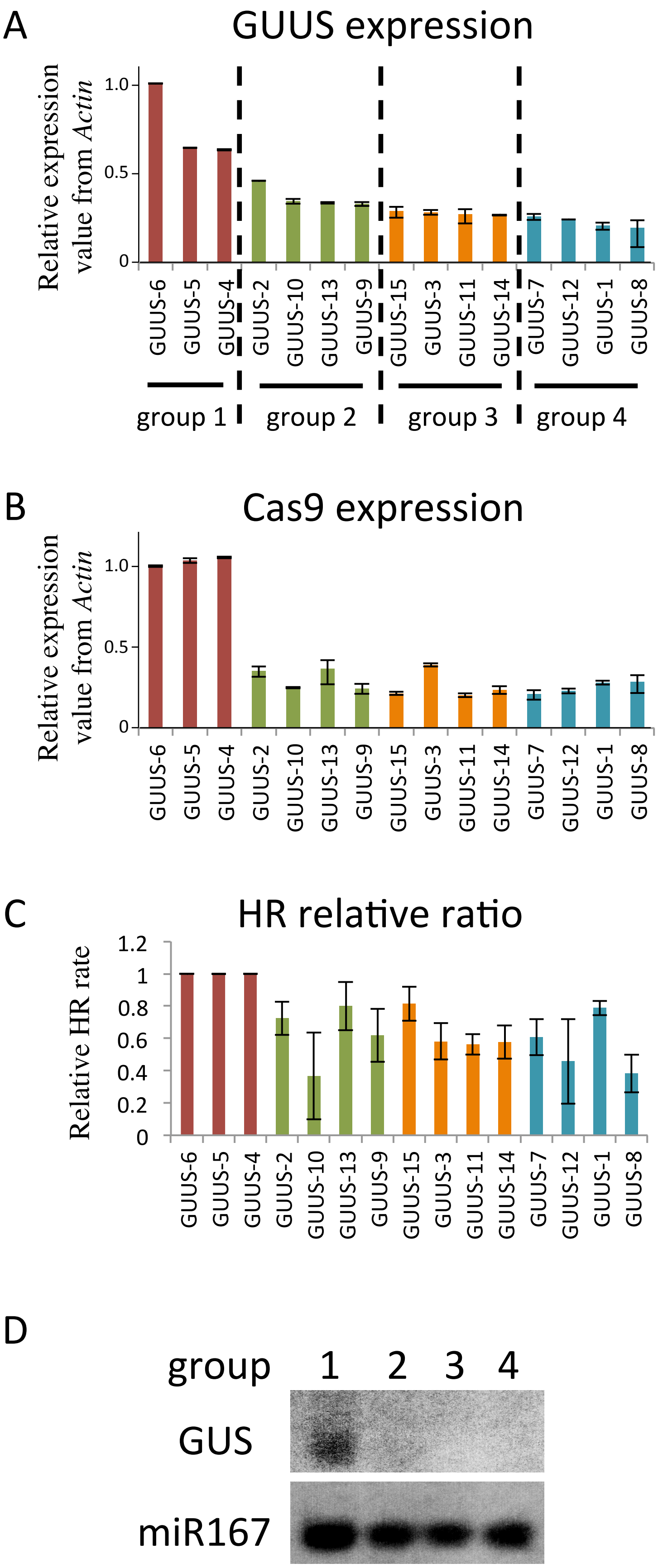

Supplementary Figure S8. relationship between GUUS expression and diRNA accumulation

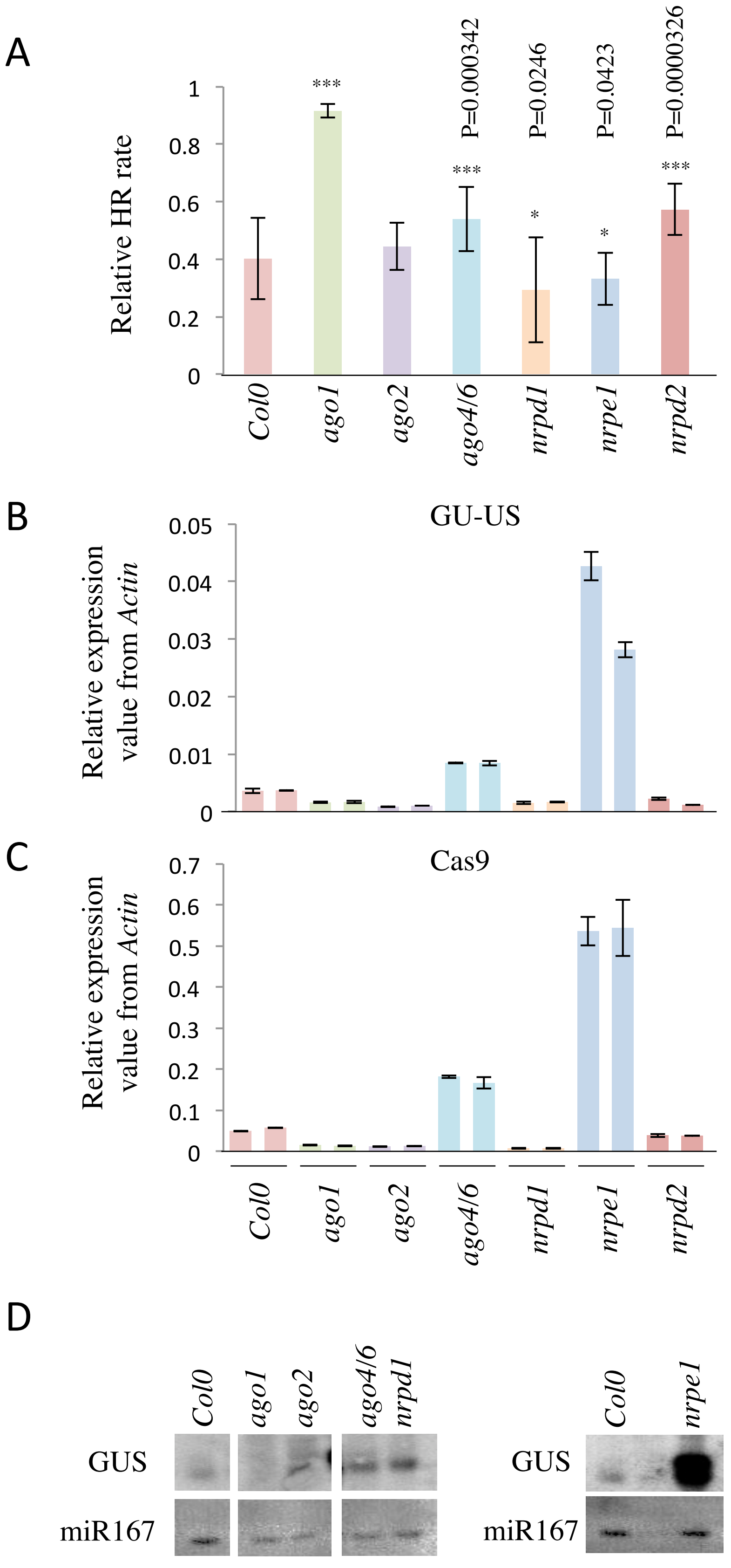

Supplementary Figure S9. GU-US repair ratio in RdDM mutants background

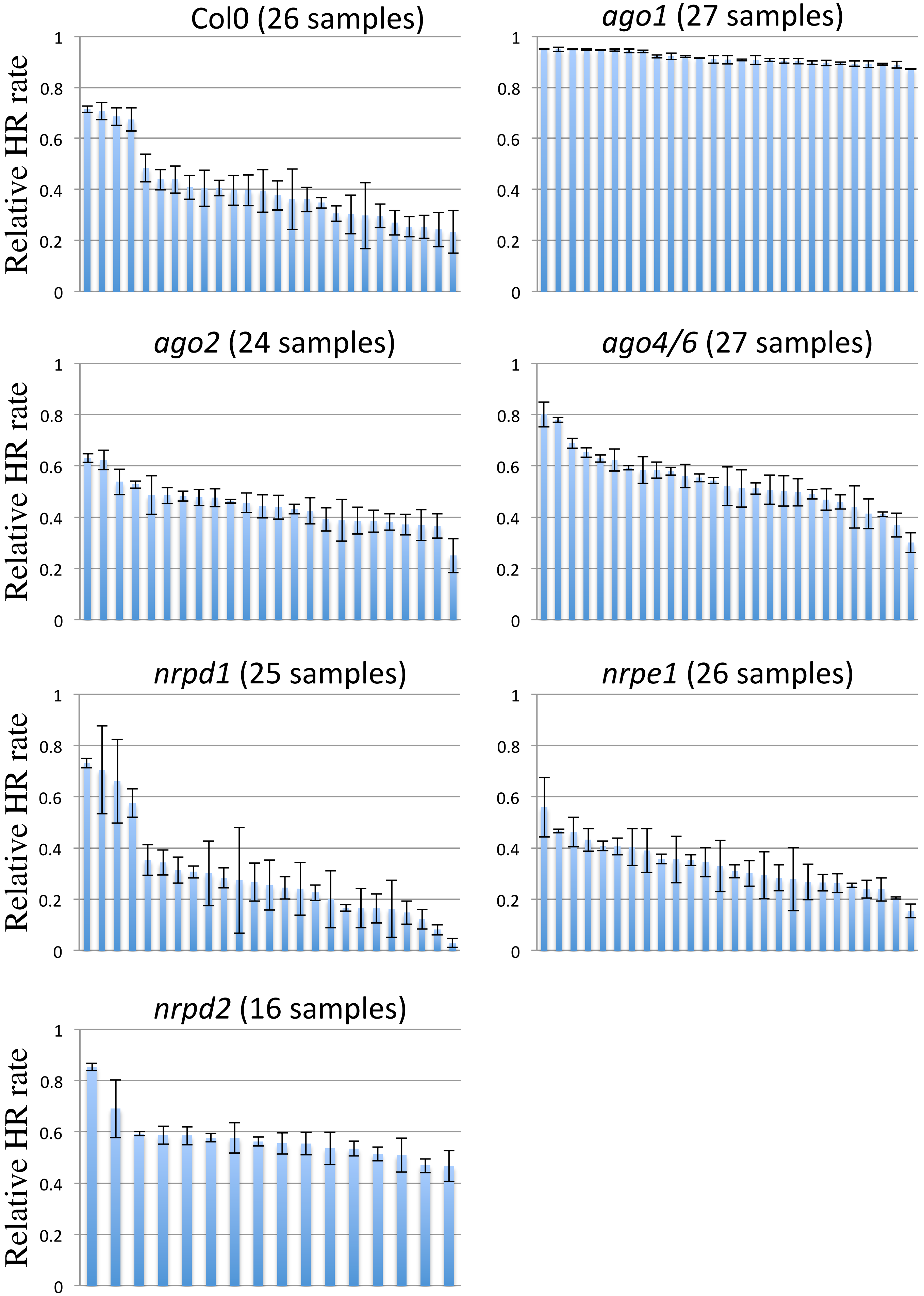

Supplementary Figure S10. GU-US HR ratio in RdDM mutants detected by qPCR

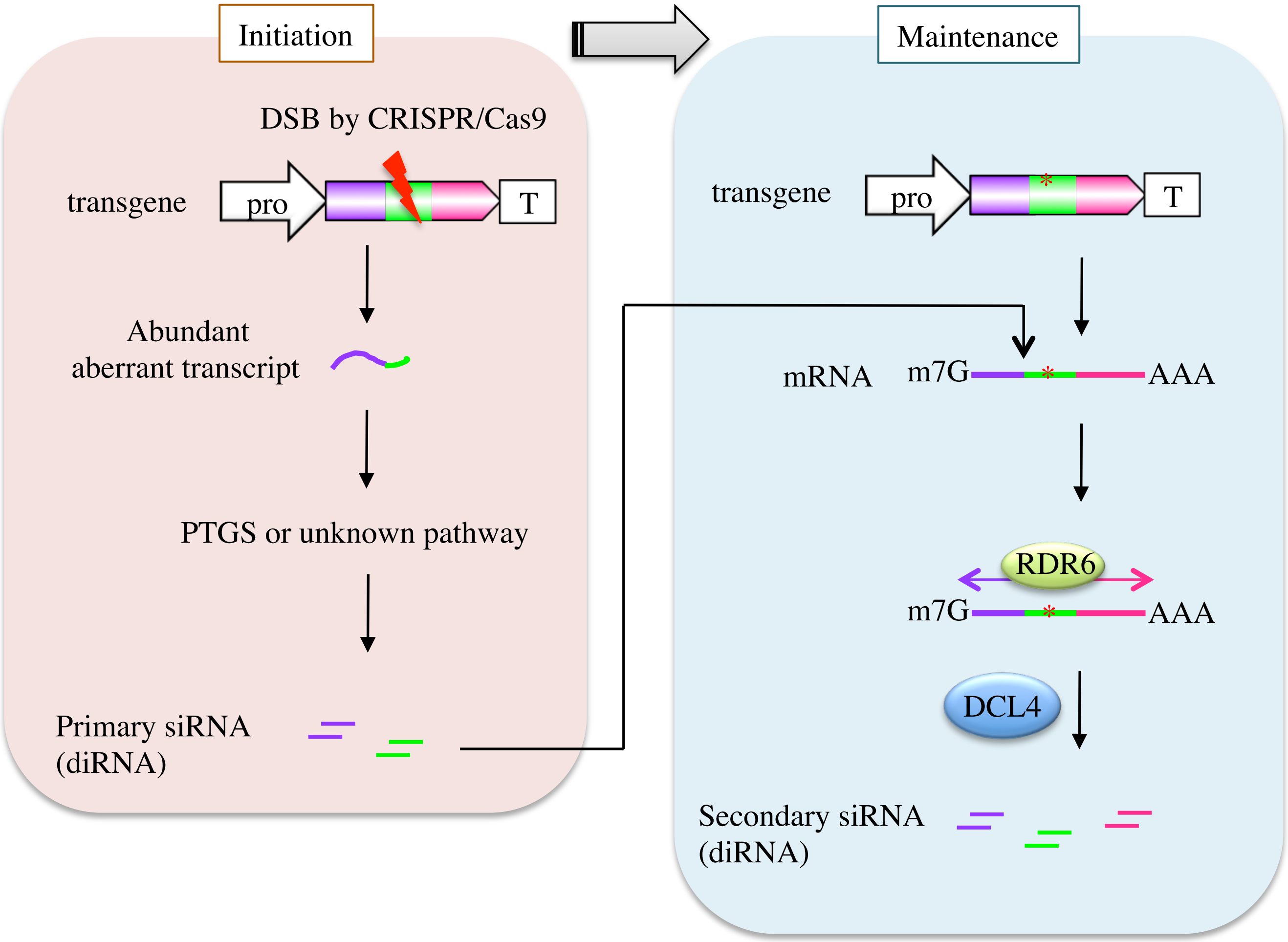

Supplementary Figure S11. Working model for diRNA production

Supplementary Information

Figure legends for Supplemental Figures

Supplementary Figure S1. Small RNA Northern blot for the 35S::GU-US transgene in *Arabidopsis* and rice. (A) Schematic representation of the GU-US reporter system and probes for Northern blotting. (B) Detection of small RNAs by Northern blot in *Arabidopsis* in T1 to T5 generation. The same membrane of Fig. 1D was re-hybridized with the indicated probe. miR167 was probed as loading control. (C-D) GU-US HR repair was determined in T0 transgenic rice by PCR (C) and qPCR (D). The primers for qPCR and calculation were same as Fig. 1C. Error bars indicate standard deviation of 3 repeats. (E) Detection of small RNAs by Northern blot in rice T0 generation.

Supplementary Figure S2. Deep sequencing of small RNAs for *AtBRI1* targeted CRISPR/Cas9 T1 transgenic lines. Three independent CRISPR/Cas9 gRNA target sites for *AtBRI1* were designed. Two biological repeats of deep sequencing for each T1 transgenic lines were performed. The y axis represents the number of mapped small RNA reads in each sample. The red, green and black boxes indicate gRNA target sites

and its neighbor regions, respectively. The sequencing results indicated that statistically there were not more small RNA signals in *AtBRI1* targeting T1 CRISPR/Cas9 lines than in WT.

Supplementary Figure S3. Antisense transcript analysis by qRT-PCR. (A-D) Detection of antisense transcripts in CRISPR/Cas9 transgenic lines by qRT-PCR. The "as" indicates antisense transcript. IGN33 was performed for Pol V transcript control. Black bar; Col0 with RT, gray bar; CRIPR/Cas9 transgenic lines with RT, white bar; without RT control. N=3. N.D.; not detected. (A) *AtGL2*, (B) At5g36250, (C) At2g36490, (D) At5g04560 targeted CRISPR/Cas9 T1 transgenic lines, respectively. Three independent transgenic lines were analyzed as biological repeats.

Supplementary Figure S4. CRISPR/Cas9 targeting to endogenous repeat regions in the At1g31290 gene. Repeat region 1 and 2 represented as green and purple arrowheads, respectively. Repeat region 1 consisted of 5x 57 bp repeats, and two gRNA1 target sites are represented by red letters. Repeat region 2 consisted of 10x 24 bp repeats, and two

gRNA2 target sites are represented by red letters.

Supplementary Figure S5. CRISPR/Cas9 targeted to the endogenous repeat region in At5g54700. The repeat region consisted of 4x 72 bp repeats, and two gRNA target sites are represented by light blue letters.

Supplementary Figure S6. Detection of antisense transcript from At5g54700. The “as” indicates antisense transcript. IGN33 was performed as a control. Blue bar; Col0 and CRIPR/Cas9 transgenic lines with RT, red bar; without RT control. N=3.

Supplementary Figure S7. qPCR analysis for GU-US repair ratio. The relative repair rate was determined by qPCR. The primers for qPCR and calculation were same as Fig. 1C. Error bars indicate standard deviation of 3 repeats. The presence (+) or absence (-) of the 35S promoter and CRIPSR/Cas9 genotype are indicated. The averages of five independent transgenic lines with or without 35S promoter are shown.

Supplementary Figure S8. Relationship between 35S::GU-US expression and diRNA accumulation. (A) Relative expression of 35S::GU-US in independent T1 transgenic lines. T1 transgenic plants were divided into four groups according to these expression levels, as indicated. (B) Relative expression value of Cas9. (C) Relative HR rate determined by qPCR. The error bars indicate standard error of 3 repeats. (D) Detection of small RNAs in the four groups. The U region of the GUS gene was used as a probe. miR167 was probed as loading control.

Supplementary Figure S9. GU-US repair ratio in RdDM mutant backgrounds. (A) Relative HR rate determined by qPCR. The error bars indicate standard error of 16-27 independent F1 plants. See also Supplemental Figure 10. Welch's t-test was performed, and asterisks indicate significant difference relative to Col0 ($p < 0.05$; *, $p < 0.01$; ***). (B-C) Relative expression of GU-US (B) and Cas9 (C) determined by qRT-PCR in RdDM mutants. (D) Northern blot detection of small RNA. The U part of GUS gene was used as a probe. miR167 was probed as loading control.

Supplementary Figure S10. GU-US HR ratio in RdDM mutants detected by qPCR. The relative repair rates in RdDM mutant backgrounds were determined by qPCR. One blue bar indicates one plant, and error bars indicate standard deviation of 3 experimental repeats.

Supplementary Figure S11. Working model for diRNA production. In this model, DSBs, induced by CRISPR/Cas9 or a restriction endonuclease, trigger transcription of aberrant RNAs. Primary siRNAs/diRNA (purple and green) generated by PTGS or unknown machinery from aberrant RNA recognize the aberrant and intact transgene mRNA, and thus promote the biogenesis of secondary siRNAs (diRNAs) (purple, green and pink).

Supplementary Table S1. Primers used in this research.
